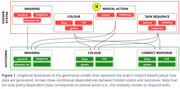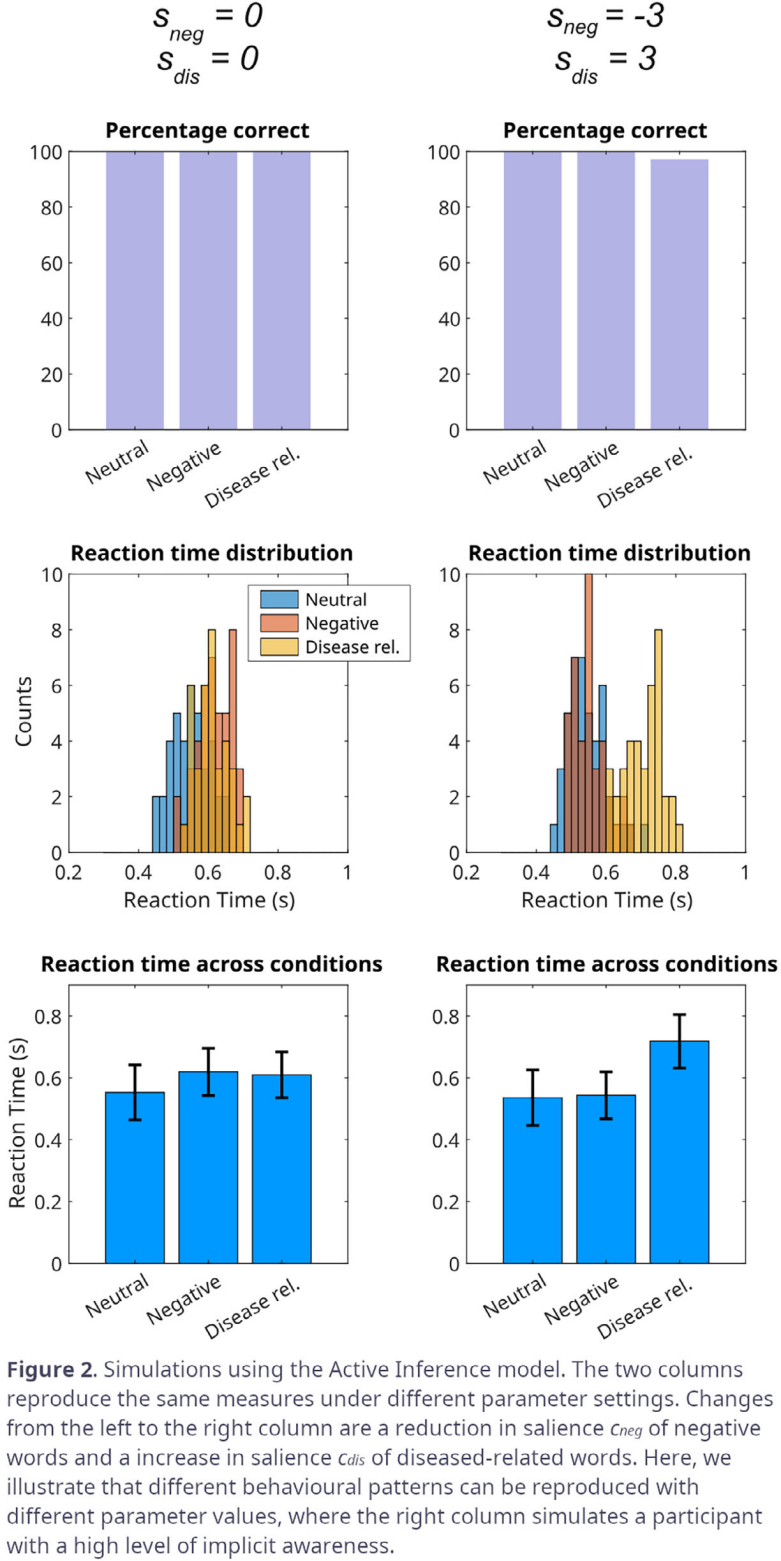# Implicit awareness in Alzheimer's Disease: an Active Inference model

**DOI:** 10.1002/alz70857_103031

**Published:** 2025-12-24

**Authors:** Daniela Ballotta, Riccardo Maramotti, Thomas Parr, Chiara Carbone, Najara Iacovino, Manuela Tondelli, Giuseppe Pagnoni, Giovanna Zamboni

**Affiliations:** ^1^ Università di Modena e Reggio Emilia, Modena, Italy; ^2^ University of Oxford, Oxford, United Kingdom; ^3^ Neurologia, Azienda Ospedaliero Universitaria di Modena, Modena, Italy

## Abstract

**Background:**

Anosognosia (i.e. lack of illness awareness) is common in neurodegenerative diseases and involves an inability to recognize cognitive, emotional, and behavioral impairments. Despite this, implicit knowledge may still influence behavior at a preconscious level (Mograbi & Morris, 2013). This can be investigated using the Emotional Stroop task, a color‐naming test that assesses interference from emotionally charged words (Martyr et al., 2011). Our previous study (Tondelli et al., 2022) showed that Alzheimer's Disease (AD) patients lacking explicit awareness exhibited distinct activation patterns in the Posterior Cingulate Cortex when processing dementia‐related words. However, reaction times varied widely, limiting traditional statistical analyses. To address this, we employed a generative model based on Active Inference, which integrates perception and action as an inferential process (Parr et al., 2022). Here, we present predictive simulations assessing the model's validity.

**Method:**

A computerized Emotional Stroop task was administered to 37 healthy controls (age 65.4 ± 6.6) and 40 AD patients (age 72.3 ± 6.7). Participants viewed neutral, negative, and disease‐related words in different colors and identified the color via button press. The experiment included 216 randomized trials, with a maximum response time of 1400 ms and a 600 ms intertrial interval. We developed a Partially Observable Markov Decision Process (POMDP) model within the Active Inference framework, incorporating four hidden state factors: word meaning, color, mental action, and task sequence (Figure 1). Four subject‐specific parameters were included: one for learning effects, a temperature term for random variability, and two for the salience of *negative* and *disease‐related* words.

**Result:**

Simulated reaction times were non‐normally distributed, mirroring the observed data. A specific slowing of reaction times for disease‐related words compared to negative words, which we assume indicate implicit knowledge of the disease, could be obtained by coupling a high value for the parameter associated with the salience of *disease‐related* words with a low value of the salience of *negative* words (Figure 2).

**Conclusion:**

Thus, the model successfully reproduced behavioral patterns, and its estimates could be linked to MRI and neuropsychological measures, potentially enabling an implicit metacognitive assessment of anosognosia.